# Gene expression response to a nematode parasite in novel and native eel hosts

**DOI:** 10.1002/ece3.5728

**Published:** 2019-10-21

**Authors:** Seraina E. Bracamonte, Paul R. Johnston, Michael T. Monaghan, Klaus Knopf

**Affiliations:** ^1^ Leibniz‐Institute of Freshwater Ecology and Inland Fisheries Berlin Germany; ^2^ Berlin Center for Genomics in Biodiversity Research Berlin Germany; ^3^ Faculty of Life Sciences Humboldt‐Universität zu Berlin Berlin Germany; ^4^ Institut für Biologie Freie Universität Berlin Berlin Germany

**Keywords:** *Anguilla anguilla*, *Anguilla japonica*, *Anguillicola crassus*, comparative transcriptomics, emerging infectious disease, host‐parasite interaction

## Abstract

Invasive parasites are involved in population declines of new host species worldwide. The high susceptibilities observed in many novel hosts have been attributed to the lack of protective immunity to the parasites which native hosts acquired during their shared evolution. We experimentally infected Japanese eels (*Anguilla japonica*) and European eels (*Anguilla anguilla*) with *Anguillicola crassus*, a nematode parasite that is native to the Japanese eel and invasive in the European eel. We inferred gene expression changes in head kidney tissue from both species, using RNA‐seq data to determine the responses at two time points during the early stages of infection (3 and 23 days postinfection). At both time points, the novel host modified the expression of a larger and functionally more diverse set of genes than the native host. Strikingly, the native host regulated immune gene expression only at the earlier time point and to a small extent while the novel host regulated these genes at both time points. A low number of differentially expressed immune genes, especially in the native host, suggest that a systemic immune response was of minor importance during the early stages of infection. Transcript abundance of genes involved in cell respiration was reduced in the novel host which may affect its ability to cope with harsh conditions and energetically demanding activities. The observed gene expression changes in response to a novel parasite that we observed in a fish follow a general pattern observed in amphibians and mammals, and suggest that the disruption of physiological processes, rather than the absence of an immediate immune response, is responsible for the higher susceptibility of the novel host.

## Graphical abstract

We inferred gene expression response in the native and a novel eel host of the parasite *Anguillicola crassus* during the early stages of infection using RNA‐seq of head kidney tissue. The native host regulated immune genes only to a small extent while the novel host regulated them more strongly in addition to genes involved in cell respiration and kidney function. Our results suggest that the disruption of physiological processes, rather than the absence of an early systemic immune response, may contribute to the higher susceptibility of the novel host.

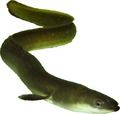

## 1 INTRODUCTION

The introduction of nonnative parasites into foreign habitats has exposed them to novel host species and has promoted several cases of disease emergence (Daszak, Cunningham, & Hyatt, 2000; Dobson & Foufopoulos, 2001; Peeler, Oidtmann, Midtlyng, Miossec, & Gozlan, 2011). Novel hosts can be highly susceptible, that is, suffer from high infection intensities (number of parasites per infected host), severe pathologies, and high fitness costs. Novel infections are leading to population declines and local extinctions of species worldwide (Peeler et al., 2011). The fungal parasites causing chytridiomycosis in amphibians and white‐nose syndrome in North American bats have led to population collapses (Frick et al., 2010; Skerratt et al., 2007), and the parasitic mite *Varroa destructor* is a major driver of honey bee declines (Le Conte, Ellis, & Ritter, 2010). The increased susceptibility that has been observed in some novel hosts may be due to a lack of defence mechanisms which the native hosts had acquired during their shared evolutionary history with the parasite (Mastitsky, Karatayev, Burlakova, & Molloy, 2010; Peeler et al., 2011).

**Figure 1:**
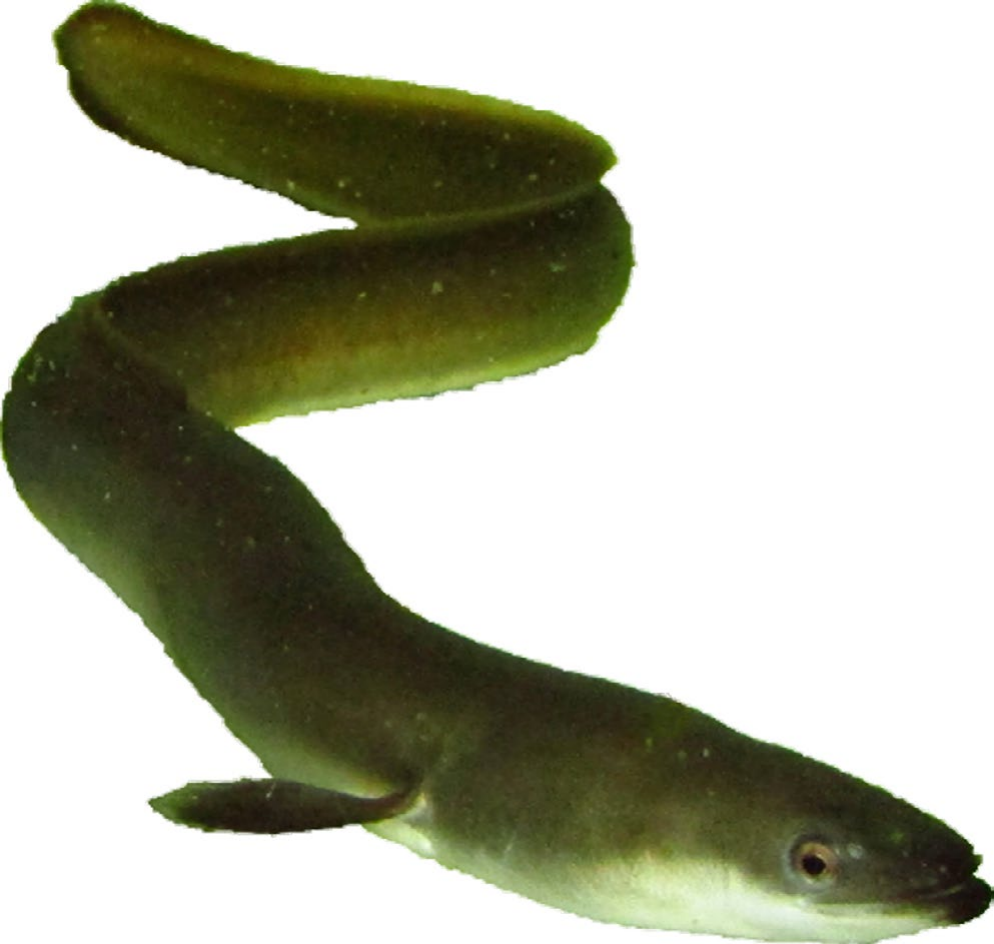
The Japanese eel (*Anguilla japonica*) is the native host of *Anguillicola crassus*, a parasitic swim bladder nematode invasive in the European eel (*Anguilla anguilla*)

A number of species of eels are threatened (Jacoby et al., 2015), and nonnative parasites in their freshwater habitat have been proposed as a contributing factor in their decline (Drouineau et al., 2018; Miller, Feunteun, & Tsukamoto, 2016; Sures & Knopf, 2004). The parasitic swim bladder nematode *Anguillicola crassus* Kuwahara, Niimi & Hagaki, 1974 was introduced into Europe from Southeast Asia where it is native to the Japanese eel (*Anguilla japonica* Temminck & Schlegel, 1846; Figure 1). It was first detected in wild European eels (*Anguilla anguilla* L., 1758) in 1982 and has rapidly spread across most of the European eel's distribution range (Kirk, 2003). In the mid‐1990s, *A. crassus* was also introduced into the American eel (*Anguilla rostrata* Lesueur, 1817) population (Barse & Secor, 1999). The parasite's introduction into Europe coincides with the onset of a steep decline of the European eel population to recruitment levels <10% of its pre‐1980 level (Bornarel et al., 2017; Diekmann, Simon, & Salva, 2019; ICES, 2018).

Natural *A. crassus* infections have not been observed to reduce body condition in the European eel (Lefebvre, Fazio, Mounaix, & Crivelli, 2013) or to affect its physiological status (Kelly, Kennedy, & Brown, 2000). However, increased stress and mortality have been reported in parasitized European eels that experienced periods of hypoxia (Gollock, Kennedy, & Brown, 2005; Lefebvre, Contournet, & Crivelli, 2007; Molnár, Székely, & Baska, 1991), indicating a cumulative negative effect from multiple stressors. Natural and experimental *A. crassus* infections impair swim bladder function (Würtz, Taraschewski, & Pelster, 1996), and laboratory swimming trials indicated that natural infections increase energy consumption and alter swimming behavior of the European eel and may thus interfere with the spawning migration and reproduction (Newbold et al., 2015; Palstra, Heppener, Ginneken, Székely, & Thillart, 2007; Pelster, 2015; Würtz et al., 1996). For the Japanese eel, body condition was not affected by *A. crassus* infections (Han et al., 2008). No data are available on how infection interacts with environmental stress in the Japanese eel, or whether *A. crassus* affects swimming, energy budget, or fitness.

A number of studies have concluded that *A. crassus* infects the European eel more successfully than the Japanese eel. In natural infections of field‐caught yellow eels (the continental freshwater feeding stage of the life cycle), infection intensities and parasite prevalence (proportion of infected hosts) were higher in the European eel (Audenaert, Huyse, Goemans, Belpaire, & Volckaert, 2003; Gérard et al., 2013; Knopf, 2006) than in the Japanese eel (Han et al., 2008; Heitlinger, Laetsch, Weclawski, Han, & Taraschewski, 2009; Münderle et al., 2006). Several infection experiments with European sources of *A. crassus* have also reported higher infection intensities in the European eel compared with the Japanese eel 12 or more weeks postinfection (Knopf & Lucius, 2008; Knopf & Mahnke, 2004). Weclawski et al. (2013) found the Japanese eel to be more efficient at killing the parasite than the European eel over the course of infection, that is past 50 days postinfection (dpi), although the infection intensity was higher in the Japanese eel at an early stage (25 dpi) when adult parasites start appearing. Earlier stages of infection have not yet been comparatively studied.

The differences in infection intensities that have been observed several weeks after infection have led to the assumption that the Japanese eel produces a more effective immune response (Knopf, 2006; Taraschewski, 2006); however, evidence for this is scarce. Both eel species develop an antibody response to adult *A. crassus* antigens, but there is no indication that the antibody response is associated with protection (Knopf & Lucius, 2008; Nielsen, 1999). In naturally infected European eels, inflammation and immune cells surrounding parasite larvae have been observed in swim bladders containing both larvae and adults (van Banning & Haenen, 1990; Molnár, Baska, Csaba, Glávits, & Székely, 1993; Würtz & Taraschewski, 2000). In natural infections, encapsulated larvae can be found at similar proportions in the swim bladder walls of both species (Audenaert et al., 2003; Heitlinger et al., 2009). High infection pressure leads to a massive increase in parasite encapsulation in the Japanese eel (Heitlinger et al., 2009).

Studies of the European eel using RNA‐seq to examine gene expression have found that genes involved in an immune response were differentially expressed in the swim bladder (the site of infection) of naturally infected eels (i.e., containing parasites of all stages) (Schneebauer, Dirks, & Pelster, 2017), as well as in the spleen and the head kidney (immune organs) very soon after experimental infection (3 dpi, i.e., containing only larvae) (Bracamonte, Johnston, Knopf, & Monaghan, 2019). Differential regulation of processes associated with both the innate and the adaptive immune system in immune organs and at the site of infection is a common feature in natural and experimental infections in vertebrates (e.g., Alvarez Rojas et al., 2015; Babayan et al., 2018; Huang et al., 2016) and the gradual shift from the regulation of innate to adaptive immune processes can be observed in gene expression studies (Ehret, Spork, Dieterich, Lucius, & Heitlinger, 2017). Additionally, parasite infections cause differential expression of genes not directly related to an immune response, such as those involved in metabolic processes, tissue repair, or organ function and development (Alvarez Rojas et al., 2015; Babayan et al., 2018; Ronza et al., 2016; Zhang et al., 2017) and this has also been observed in the European eel (Bracamonte et al., 2019; Schneebauer et al., 2017). How *A. crassus* affects gene expression in the Japanese eel and what processes are modified upon infection have not yet been determined for any parasitic stage.

RNA‐seq studies of infection experiments on a range of species indicate that the number of affected processes, the magnitude of change, and the specific genes involved differ considerably among host species‐parasite species systems (e.g., Alvarez Rojas et al., 2015; Haase et al., 2016; Kumar, Abd‐Elfattah, & El‐Matbouli, 2015; Zhang et al., 2017). Infections with invasive parasites have consistently induced a more pronounced response in susceptible hosts compared with resistant hosts. In both frogs and toads suffering from chytridiomycosis, a larger number of genes were differentially expressed in susceptible species and they were involved in a more diverse set of processes, including several immune‐related and metabolic processes (Eskew et al., 2018; Poorten & Rosenblum, 2016). Similar patterns were observed in bats exposed to the fungus causing white‐nose syndrome (Davy et al., 2017; Field et al., 2015) although the resistant species had cleared the infection at the time of sampling. Finally, more genes were differentially expressed in a more susceptible bee species exposed to *Varroa* mites (Zhang, Liu, Zhang, & Han, 2010), although the diversity of processes was not reported. For the eel‐*Anguillicola* host‐parasite system, the processes leading to the different outcome of infection between eel species and the seemingly larger impact on the European eel are still unknown but they may also result from this emerging general pattern.

Here, we experimentally infected Japanese eels and European eels with *A. crassus* under controlled conditions. We measured the number of parasites in swim bladders and used RNA‐seq to estimate gene expression changes in the head kidney at two time points in the early stages of infection: during the migrating phase of the larval parasite (3 dpi) and after the establishment of larvae in the swim bladder (23 dpi). Our main goal was to test whether processes modified during the early stages of infection contribute to the higher susceptibility of the European eel, the novel host, compared with the Japanese eel, the native host. We also tested whether European eels differentially express a larger number of genes and greater diversity of processes, as potentially predicted by recent observations in amphibians and mammals. If novel hosts produce an ineffective immune response, we expected that maintaining homeostasis, such as metabolism and renal function, would be more problematic for the European eel than for the Japanese eel and that changes in gene expression would be the result.

## 2 MATERIALS AND METHODS

### 2.1 Experimental setup and sampling

Japanese eels were imported as glass eels (transition from marine larval stage to freshwater stage) from Japan in 2006 and raised to the yellow eel stage in the laboratory at the Leibniz‐Institute of Freshwater Ecology and Inland Fisheries (Berlin, Germany). The eels have never been exposed to *A. crassus*. European eels were purchased as yellow eels in 2004 from an eel farm in Germany (Domäne Voldagsen, Einbeck) that was free of *A. crassus*. Thereafter, both species were kept in recirculation systems in aerated tap water. Each individual was housed in a separate compartment (40–80 L) within larger aquaria (200 L). Each compartment contained a polyethylene tube for hiding.

The parasite *A. crassus* is trophically transmitted, and anguillid eels are the only known final hosts (De Charleroy, Grisez, Thomas, Belpaire, & Ollevier, 1990; Nagasawa, Kim, & Hirose, 1994). Free‐living second stage larvae (L_2_) hatch from eggs in fresh water and are consumed by crustacean plankton (Copepoda), the intermediate hosts. In the copepod, they molt into third‐stage larvae (L_3_) which is the infective stage for eels. Eels are infected by feeding on intermediate or paratenic hosts. *A. crassus* L_3_ migrate from the intestine to the swim bladder wall in approximately one week (Haenen, Grisez, Decharleroy, Belpaire, & Ollevier, 1989; Knopf, Würtz, Sures, & Taraschewski, 1998). Two to 3 weeks postinfection, they molt into fourth stage larvae (L_4_). At 25 dpi, adults can be present in the swim bladder lumen (Weclawski et al., 2013). They reproduce sexually, and the eggs are released into the water.

Infection of eels with *A. crassus* was carried out at the beginning of the experiment following the method of Knopf et al. (1998). In short, *A. crassus* eggs were collected from the swim bladder of wild European eels caught from nearby Lake Müggelsee (Berlin, Germany) in autumn 2014. The L_2_ were hatched and fed to copepods from the same lake. Three weeks postinfection, the copepods were crushed to extract L_3_ that were then suspended in phosphate‐buffered saline (PBS, pH 7.2). For both eel species, 10 individuals were infected with 25 L_3_ individuals suspended in 100 μl PBS using a stomach tube, while nine Japanese eels and 10 European eels were sham‐infected with 100 μl PBS and served as controls. At 3 dpi, five control and five infected individuals of each species were dissected and the head kidney was removed and stored in RNA*later* (Life Technologies, Darmstadt, Germany) at −20°C. The remaining nine Japanese eels (5 infected and 4 control) and 10 European eels (5/5) were dissected at 23 dpi, and the head kidney was stored in RNA*later* at −20°C or flash‐frozen in liquid nitrogen and stored at −80°C until processing. For all individuals, the swim bladder was removed during dissection and checked for the presence of *A. crassus* under a binocular. *Anguillicola crassus* individuals were counted, and their developmental stage was determined (L_3_ and L_4_). No adult *A. crassus* were present at any sampling time. At the time of dissection, all individuals were weighed and measured. The sex was not determined, because gonads of eels are of an undifferentiated state and cannot be determined microscopically until eels reach an advanced stage of maturity during the spawning migration (Tesch, 2003). The Berlin State Office for Health and Social Affairs (LaGeSo) in Germany approved the experimental procedure (approval number G 0021/15).

### 2.2 RNA extraction and sequencing

RNA of European eel samples collected at 3 dpi was extracted and sequenced as described by Bracamonte et al. (2019). Japanese eel samples collected at 3 dpi and 2 control and 2 treatment samples of the European eel collected at 23 dpi were stored in RNA*later*. The remaining six samples of the European eel collected at 23 dpi and all Japanese eel samples collected at 23 dpi were shock‐frozen in liquid nitrogen and stored at −80°C. Storage condition was included as a factor in the relevant model (see below). RNA was extracted with TRIzol (Life Technologies) following the manufacturer's recommendations for fatty tissue with slight modifications. A TissueLyzer II (Eppendorf) was used to homogenize tissue in 850 μl TRIzol. After centrifugation, another 150 μl TRIzol and 200 μl chloroform were added to the supernatant. RNA was precipitated with 500 μl isopropanol and washed with 1 ml 75% ethanol. It was resuspended in 50 μl DEPC‐water (Life Technologies) and incubated on a heat block at 50°C for 2 min. Concentrations were measured with a 2100 Bioanalyzer (Agilent Technologies). Samples were diluted to 40 ng/μl in DEPC‐water, precipitated with 3 M sodium acetate and 100% ethanol. Library preparation and paired‐end sequencing (100 bp) were performed at Macrogen on an Illumina HiSeq4000. The number of raw reads per sample ranged from 14.6 to 30.8 M (Dryad Repository).

### 2.3 Data analysis

Differences in length and weight between the two eel species were assessed using Wilcoxon rank‐sum tests in R v.3.3.2 (R Core Team, 2016). Wilcoxon rank‐sum tests were also used to determine differences in infection intensities, that is, number of larvae in the swim bladder, between the two species within sampling days (3, 23 dpi).

Reads were de novo assembled into one transcriptome per species with Trinity v2.3.1 prerelease (Grabherr et al., 2011; Haas et al., 2013) using Bowtie v.1.1.2 (Langmead, Trapnell, Pop, & Salzberg, 2009). For the European eel, raw reads from the head kidney samples from a previous study (Bracamonte et al., 2019; NCBI BioProject accession PRJNA419718) and from this study were combined. Reads from the previous study had a Phred score >30. All reads from this study had a Phred score >20. For the European eel, 95.95–96.62% of the reads per sample had a Phred score >30. For the Japanese eel, reads with a Phred score >30 ranged between 94.93% and 96.81% per sample. For both species, Trinity was run with default parameters, including per sample and overall in silico normalization and quality trimming using the trimmomatic option. Assembly quality and statistics were calculated using the provided Trinity scripts and Bowtie2 v2.2.9 (Langmead & Salzberg, 2012). Orthologous genes in the Japanese eel and the European eel were identified with OrthoFinder v1.1.4 using default parameters (Emms & Kelly, 2015).

Annotations for both transcriptomes were derived from blastx and blastp searches against the UniProtKB/Swiss‐Prot (http://www.uniprot.org) and the RefSeq (http://www.ncbi.nlm.nih.gov/refseq/) databases. The *E*‐value cutoff was set to 0.001. Conserved domains were identified by searching the Pfam database with HMMER v3.2.1 (http://hmmer.org/). Annotations obtained from RefSeq were examined for their taxonomic composition and contigs that best matched bacterial sequences were removed from the transcriptomes. For the remaining contigs, GO assignments were retrieved with Trinotate v3.2.0 from annotations obtained from Swiss‐Prot and Pfam. Differentially expressed genes without annotation were blasted against the nr database of NCBI (http://www.ncbi.nlm.nih.gov).

**Figure 2:**
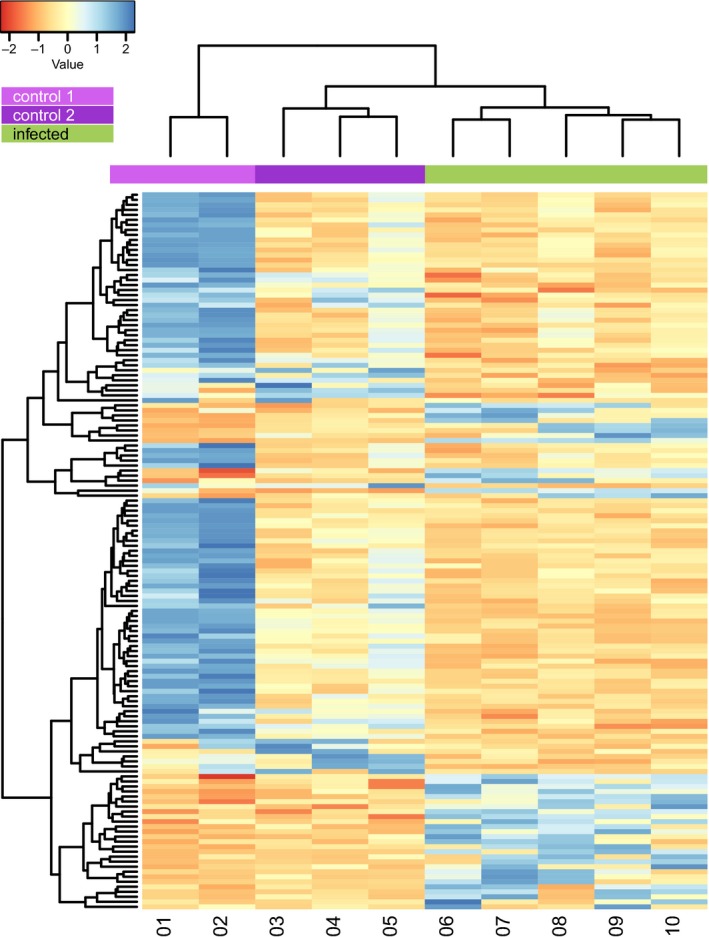
Heatmap of differentially expressed genes in the Japanese eels (*Anguilla japonica*) at 3 days postinfection (dpi) prior to correction for the control group. Each column represents one sample, and each row corresponds to a differentially expressed gene. Control samples form two distinct clusters (light and dark violet bars). Infected samples are indicated by a green bar. Red indicates reduced expression, blue indicates increased expression. The values are gene‐level *z*‐scores. The dendrogram is computed using Euclidean distances and clustered by the means

Gene expression was analyzed separately for each species and sampling day with DESeq2 (Love, Huber, & Anders, 2014). We could not analyze the two sampling days in a single model because sample processing differed between sampling days (see above). The need to control for the potentially large technical variation introduced by that (Leek et al., 2010) resulted in sample processing being confounded with the four treatment factors (control/infected at 3 dpi, control/infected at 23 dpi) and such models cannot be fit in DESeq2. Thus, we analyzed two sampling days in separate models. Gene‐level abundance estimates were calculated using RSEM v1.3.0 (Li & Dewey, 2011). The read alignment rate for each sample ranged from 64.2% to 75.6% (Dryad Repository). Abundance estimates were modeled using generalized linear models of the negative binomial family with a logarithmic link using DESeq2 v1.14.0 (Love et al., 2014) after removing contigs with low coverage (mean coverage <10; Todd, Black, & Gemmell, 2016). Hereafter, we refer to contigs that were maintained for differential gene expression analyses as genes. For the Japanese eel, treatment (infected, control) was included as a factor in the model for 3 dpi and in the model for 23 dpi. For the European eel, the model for 3 dpi included treatment as well as sequencing batch, the latter to control for the fact that sequencing was performed on different plates (see Bracamonte et al., 2019). The model for 23 dpi for the European eel included storage condition and treatment to control for the two sample storage conditions (see above). For all analyses, dispersion parameters were estimated with a local fit. Empirical Bayes shrinkage was applied to both dispersions and logarithmic fold changes. Genes for which expression between treatments differed by a log_2_ fold change ≥1 with an adjusted *p*‐value <.05 were considered to be differentially expressed genes (DEG). *p*‐Value adjustment followed the Benjamini–Hochberg procedure as implemented in DESeq2 after independent filtering using the mean normalized count for each gene across all samples. At 3 dpi, Japanese eel control samples were separated into two distinct clusters based on DEG (Figure 2). If the difference in (rlog) expression between treatment and either control cluster was smaller than the difference between control clusters, that gene was not considered to be differentially expressed (see below).

Differentially expressed genes were used to estimate Gene Ontology (GO) enrichment with GOstats v2.48.0 (Falcon & Gentleman, 2007). GO assignments obtained with Trinotate were used as a reference. Enrichment analysis was restricted to the domain “biological processes.” Conditional hypergeometric tests were performed with a *p*‐value cutoff of .01. We only calculated overrepresentation of GO terms. Overrepresented GO terms were summarized with the web application CateGOrizer (Hu, Bao, & Reecy, 2008) using GO classifications available from CateGOrizer, but excluding the three general terms “metabolism,” “immunology, immune response,” and “response to stress.” First, the GO classification “Immune system gene classes” was used on all overrepresented GO terms, then “GO_Slim2” was used for GO terms that could not be summarized by immune classes, lastly, the three general terms were used on GO terms that could not be summarized by the two GO classification lists.

## 3 RESULTS

### 3.1 Experimental infection

**Figure 3:**
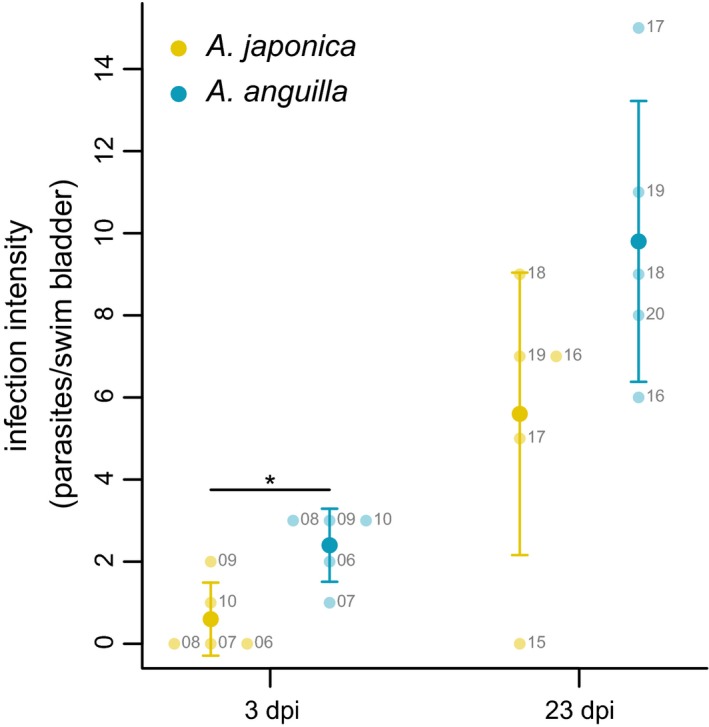
Mean infection intensities (±*SE*) for Japanese eels (*Anguilla japonica*, yellow) and European eels (*Anguilla anguilla*, cyan) at 3 days postinfection (dpi) and 23 dpi. Light‐colored dots give number of parasites/swim bladder for each individual, *Significant difference between species

Japanese eels were significantly larger (mean ± *SD* 54.9 ± 7.8 cm) than European eels (41.9 ± 3.0 cm; Wilcoxon rank‐sum test, *W* = 9, *p* < .001) and were heavier (216.8 ± 113.3 g) than European eels (113.7 ± 20.4 g; *W* = 9, *p* < .001). At 3 dpi, the infection intensity with *A. crassus* in swim bladders was greater in European eels (mean ± *SD* 2.4 ± 0.9) than in Japanese eels (0.6 ± 0.9) (Figure 3; *W* = 23, *p* = .03). Only L_3_ were recovered at 3 dpi. At 23 dpi, the mean infection intensity was higher in both species (Figure 3) but it did not differ significantly between species (Figure 3; *W* = 20.5, *p* = .12; 5.6 ± 3.4 for Japanese eels, 9.8 ± 3.4 for European eels). Both L_3_ and L_4_ were recovered from both eel species at 23 dpi. No adult or dead *A. crassus* were recovered. None of the control individuals were infected at any stage.

### 3.2 Differential gene expression analysis

For the Japanese eel, 21,748,029 reads were assembled into 255,431 contigs and 347,581 isoforms with a mean coverage of 11.25. The average length of the isoforms was 556 bp, and the N50 was 763 bp (Table 1). For the European eel, 45,528,485 reads were assembled into 508,838 contigs and 693,979 isoforms. The mean coverage was 10.76. The N50 was 910 bp, and the average isoform length was 610 bp (Table 1). RefSeq annotations revealed that ~6% of the Japanese eel contigs and ~1% of the European eel contigs correspond to bacterial contamination and they were removed from further analyses.

**Table 1:** Assembly and annotation statistics. Data for each sample are available from the Dryad Repository

	Japanese eel		European eel	
Assembled reads	21,748,029		45,528,485	
Properly paired	20,669,880	95.04%	42,944,499	94.32%
Improperly paired	728,604	3.35%	1,879,425	4.13%
Single read	349,545	1.61%	704,561	1.55%
Number of contigs	255,431		508,838	
Bacterial contamination	14,238	5.57%	5,616	1.10%
GO annotation	47,429	19.66%	56,977	11.32%
RefSeq annotation	62,358	25.85%	136,334	27.09%
Number of isoforms	347,581		693,979	
Mean coverage	11.25		10.76	
N50 (bp)	763		910	
Mean length (bp)	556		610	
GC content	46.53%		46.34%	

Note

Mean coverage, N50, and mean length refer to isoforms. Percentage of GO and RefSeq annotations refer to the clean number of contigs after removal of bacterial contamination.

Abbreviation: bp, base pairs.

**Figure 4:**
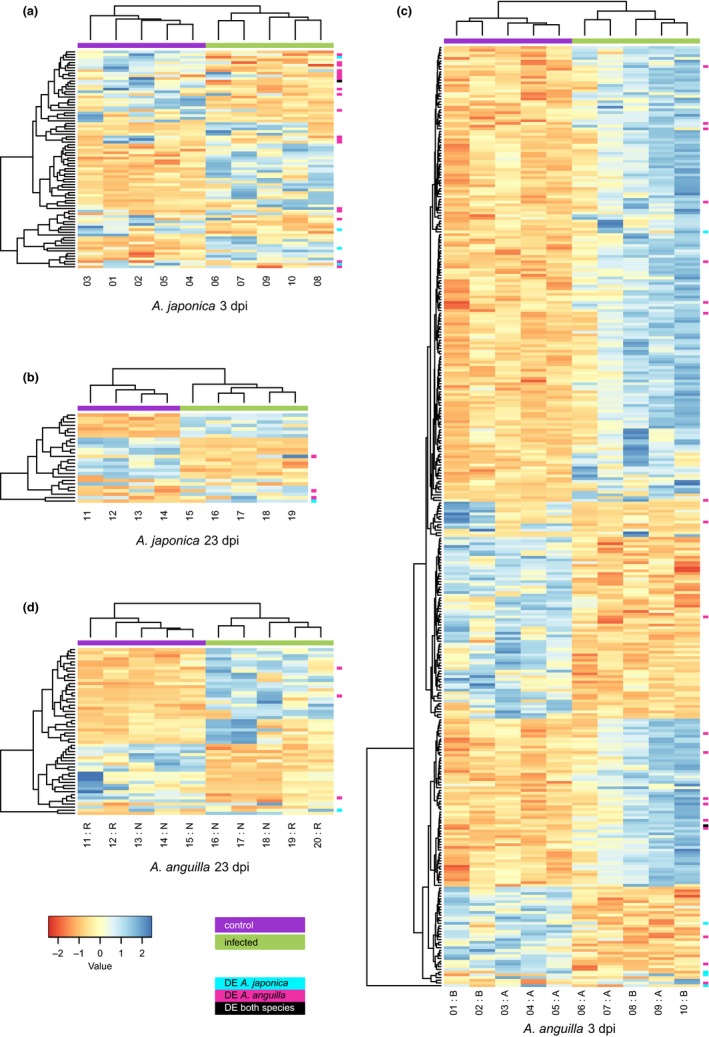
Heatmaps of significantly differentially expressed genes in the Japanese eel (*Anguilla japonica*) at (a) 3 days postinfection (dpi) and (b) 23 dpi and in the European eel (*Anguilla anguilla*) at (c) 23 dpi and (d) 3 dpi. When multiple orthologs were mapped to a differentially expressed gene in the other species, these orthologs were combined and are presented as a single row in the heatmaps. Red indicates reduced expression, blue indicates increased expression. Violet bars = control samples, green bars = infected samples. Cyan squares indicate orthologs that were differentially expressed in the Japanese eel, pink squares indicate orthologs that were differentially expressed in the European eel. For the European eel, A and B are the sequencing batches, tissue storage condition is indicated by R (RNA*later*) and N (liquid nitrogen). Gene‐level *z*‐score values are presented. Dendrograms are computed based on Euclidean distances and clustered by the means

In Japanese eels, there were 64 DEG in infected eels at 3 dpi compared with control individuals (Table 2), with log_2_ fold changes ranging from −5.19 to 6.79 (Figure 4a). This reduced to 23 DEG at 23 dpi (Table 2), with log_2_ fold changes ranging from −4.47 to 3.35 (Figure 4b). Only one gene was differentially expressed at both time points, with increased expression at 3 dpi and decreased expression at 23 dpi. Unfortunately, it was not annotated. There were considerably more DEG in infected European eels, with 342 DEG in infected eels at 3 dpi compared with control individuals (Table 2). Log_2_ fold changes ranged from −4.15 to 4.65 (Figure 4c). At 23 dpi, this reduced to 53 DEG (Table 2). Log_2_ fold changes ranged from −7.61 to 6.74 (Figure 4d). Similarly to the Japanese eel, one unannotated gene was differentially expressed at both 3 and 23 dpi, although its transcript abundance was reduced at both time points. Among the differentially expressed genes of both species and time points, 29 genes were assigned to 27 orthologous groups (Table 3, Figure 4). Log_2_ fold changes for the majority of orthologs were <1. Only one ortholog, a tripartite motif‐containing protein, was differentially expressed in both species. Its transcript abundance was reduced in the Japanese eel but elevated in the European eel (Table 3).

**Table 2:** Number of differentially expressed genes (DEG), DEG with annotations, and overrepresented Gene Ontology (GO) terms

Species	Dpi	Up‐regulated			Down‐regulated		
		Total DEG	Annotated	GO terms	Total DEG	Annotated	GO terms
Japanese eel	3	39	11	6	25	17	33
	23	11	2	0	12	1	6
European eel	3	233	139	134	209	49	41
	23	33	19	50	20	8	17

Abbreviation: dpi, days postinfection.

**Table 3:** Log2 fold changes of orthologous genes differentially expressed in either Japanese eels or European eels. If multiple orthologous genes were retrieved for one differentially expressed gene, log2 fold changes are given separately for each of them. Genes are sorted according to the clustering on the heatmap (Figure 4)

Species	UniProt or RefSeq annotation	Base mean	Log2 FC	Adj. *p*‐value	Dpi	Ortho
Japanese eel	Complement C3	22.855	−1.722	0.245	3	g1
	Sulfate transporter	**21.866**	**−2.533**	**0.049**	3	g2
	Transmembrane protein 82	8.690	−0.933	0.474	3	g3
	Secretory phospholipase A2 receptor	8.302	−1.443	0.200	3	g4
	BTB/POZ domain‐containing protein 17	9.973	−0.913	0.655	3	g5
	Collagen alpha‐5(IV) chain	14.480	0.042	0.981	3	g6
	Receptor tyrosine‐protein kinase erbB‐3	10.106	0.372	0.785	3	g7
	Ras‐related protein Rab	32.712	−1.515	NA	3	g8
	Tripartite motif‐containing protein	**49.420**	**−2.497**	**0.012**	3	g9
	Ceramide synthase 2	7.987	−2.748	0.123	3	g10^a^
	Ceramide synthase 2	10.409	−0.200	0.888	3	g10^a^
	Platelet‐derived growth factor C	31.101	0.267	0.858	3	g11
	Putative ribonuclease H protein At1g65750	26.217	−0.724	0.789	3	g12
	Neural cell adhesion molecule 1	15.284	−2.222	0.237	3	g13
	SLAM family member 5	12.054	−2.247	NA	3	g14
		16.941	−2.297	0.288	3	g15
	uncharacterized protein LOC106600263, partial	218.131	1.911	0.419	3	g16
	Wilms tumor protein homolog	93.910	0.126	0.850	3	g17
	Estrogen receptor beta	46.955	0.154	0.901	3	g18
	Transmembrane protein 150B	**274.372**	**−4.660**	**0.000**	3	g19
	Erythropoietin receptor	**1,631.368**	**4.064**	**0.010**	3	g20
	Ig lambda‐1 chain V region S43	1,099.355	−0.074	0.963	3	g21
	Olfactomedin‐4	**671.199**	**−1.665**	**0.003**	3	g22
	Cytochrome P450	76.495	−2.232	0.219	3	g23^a^
	Cytochrome P450	116.302	0.369	0.752	3	g23^a^
European eel	Wilms tumor protein homolog	**40.787**	**1.589**	**0.016**	3	g17
	Receptor tyrosine‐protein kinase erbB‐3	**15.722**	**1.607**	**0.028**	3	g7^b^
	uncharacterized protein LOC106600263, partial	**35.068**	**2.528**	**0.017**	3	g16
	Ceramide synthase 2	**26.228**	**1.969**	**0.036**	3	g10
	Tripartite motif‐containing protein	34.275	0.491	0.949	3	g9^b^
	Tripartite motif‐containing protein	73.534	−0.527	0.896	3	g9^b^
	Tripartite motif‐containing protein	62.575	−0.279	0.963	3	g9^b^
	Tripartite motif‐containing protein	100.232	−0.363	0.898	3	g9^b^
	Tripartite motif‐containing protein	22.121	−0.540	0.969	3	g9^b^
	Transmembrane protein 82	**16.591**	**2.539**	**0.004**	3	g3
	Receptor tyrosine‐protein kinase erbB‐3	**13.040**	**2.184**	**0.003**	3	g7^b^
	Ras‐related protein Rab	**18.986**	**2.448**	**0.011**	3	g8
	Putative ribonuclease H protein At1g65750	**46.092**	**3.034**	**0.024**	3	g12
	Neural cell adhesion molecule 1	**69.045**	**−2.592**	**0.025**	3	g13
	SLAM family member 5	**12.386**	**−1.902**	**0.003**	3	g14
	Estrogen receptor beta	**242.160**	**2.552**	**0.020**	3	g18
	Collagen alpha‐5(IV) chain	**88.892**	**1.837**	**0.025**	3	g6
	Complement C3	**522.554**	**2.495**	**0.036**	3	g1
		**239.109**	**1.979**	**0.045**	3	g15
	Secretory phospholipase A2 receptor	**48.205**	**1.514**	**0.018**	3	g4
	Tripartite motif‐containing protein	**59.652**	**1.609**	**0.014**	3	g9^b^
	Platelet‐derived growth factor C	**49.289**	**1.657**	**0.013**	3	g11
	Erythropoietin receptor	69.219	−0.708	0.847	3	g20
	BTB/POZ domain‐containing protein 17	**36.409**	**−2.534**	**0.000**	3	g5
	Ig lambda‐1 chain V region S43	**153.169**	**−3.156**	**0.000**	3	g21
	Olfactomedin‐4	765.320	−0.495	0.809	3	g22
	Transmembrane protein 150B	72.345	3.167	NA	3	g19
	Cytochrome P450	**816.051**	**1.866**	**0.029**	3	g23
	Sulfate transporter	586.823	0.280	0.849	3	g2
Japanese eel	Apolipoprotein A‐I‐1	8.782	0.491	NA	23	g24
	LPS‐induced TNF‐alpha factor homolog	34.972	0.316	1.000	23	g25
	Probable G‐protein coupled receptor 34	112.783	−0.204	1.000	23	g26^a^
	Probable G‐protein coupled receptor 34	37.250	0.346	1.000	23	g26^a^
	40S ribosomal protein S27‐like	**24,422.925**	**2.427**	**0.026**	23	g27
European eel	Apolipoprotein A‐I‐1	**60.270**	**5.024**	**0.001**	23	g24
	LPS‐induced TNF‐alpha factor homolog	**9.189**	**4.920**	**0.039**	23	g25
	Probable G‐protein coupled receptor 34	**76.144**	**−1.841**	**0.000**	23	g26
	40S ribosomal protein S27	19,585.355	0.118	0.962	23	g27

Note

Significantly different log2 fold changes are given in bold.

Abbreviations: Adj. *p*‐value, Benjamini–Hochberg‐adjusted *p*‐value calculated by DESeq2; Base mean, mean expression across all samples; dpi, days postinfection; Log2 FC, log2 fold expression change between infected and control samples; Ortho, orthologous groups.

a Multiple orthologous genes in the Japanese eel.

b Multiple orthologous genes in the European eel.

### 3.3 Functional analysis

**Figure 5:**
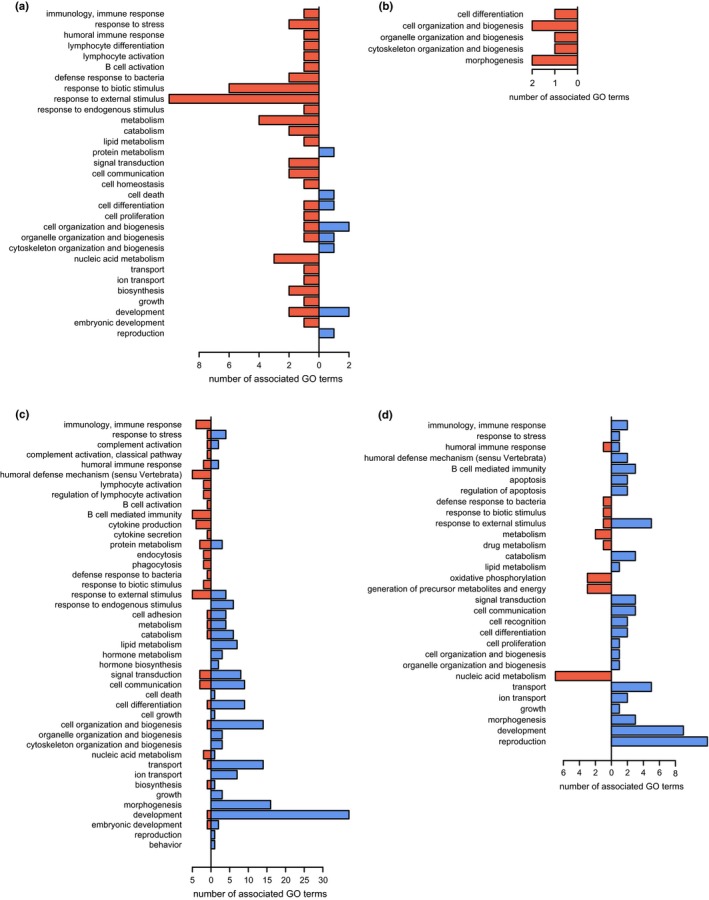
Overrepresented Gene Ontology terms categorized into immune classes and GO_slim2 in (a) the Japanese eel (*Anguilla japonica*) at 3 days postinfection (dpi) and (b) 23 dpi and the European eel (*Anguilla anguilla*) at (c) 3 dpi and (d) 23 dpi. Blue bars to the right indicate enrichment in up‐regulated genes, red bars to the left indicate enrichment in down‐regulated genes of infected individuals

For the Japanese eel, 44% of DEG at 3 dpi and 13% of DEG at 23 dpi retrieved UniProt or RefSeq annotations. This resulted in 39 overrepresented GO terms at 3 dpi and six overrepresented GO terms at 23 dpi (Table 2). Most GO terms were down‐regulated and associated with metabolic and cellular GO_slim2 categories (Figure 5a,b). At 3 dpi, processes related to an immune response were overrepresented among the down‐regulated genes (Dryad Repository). These included antimicrobial response and negative regulation of TLR signaling. Transcript abundance of an immunoglobulin (Ig) lambda‐like polypeptide was elevated and the abundance of *cd40* transcripts, which leads to Ig secretion via activation of B cells by T cell costimulation, was reduced (Dryad Repository). The abundance of erythropoietin receptor transcripts was elevated. At 23 dpi, the overrepresented GO terms for down‐regulated genes were related to muscle contraction. For the up‐regulated genes, there were no overrepresented GO terms (Table 2, Dryad Repository). The full lists of overrepresented GO terms and DEG can be found on the Dryad Repository.

For the European eel, annotations were retrieved for 55% of DEG at 3 dpi and 51% of DEG at 23 dpi. There were 175 overrepresented GO terms at 3 dpi and 67 overrepresented GO terms at 23 dpi (Table 2). Unlike for the Japanese eel, GO terms were mostly up‐regulated at both time points. Categories of immune system gene classes and GO_slim2 assigned them to a more diverse set of categories than the GO terms of the Japanese eel. Notably, more immune system categories were assigned for the European eel (Figure 5c,d). At 3 dpi, GO terms associated with an immune response were overrepresented in both up‐and down‐regulated genes (Dryad Repository). The regulation of cytokines, phagocytosis, and B cell activation was overrepresented in the down‐regulated genes while terms related to arachidonic acid and icosanoid secretion as well as complement activation were overrepresented in the up‐regulated genes. Furthermore, transcript abundance of genes associated with wound healing was elevated. The abundance of major histocompatibility complex (MHC) class IIA and T cell receptor (TCR) α transcripts, both of which are essential for an adaptive immune response, was reduced at 3 dpi, as was the transcript abundance of six Ig chains (Dryad Repository). Genes involved in cell adhesion and motility were overrepresented among the up‐regulated genes, as well as genes involved in renal development and function (Dryad Repository). Transcript abundance of oxygen‐dependent coprophorphyrinogen‐III oxidase (*cpox*), an enzyme involved in heme biosynthesis, was reduced. The abundance of N‐acetyl‐D‐glucosamine kinase (*nagk*) transcripts, involved in amino sugar metabolism, was also reduced (Dryad Repository). At 23 dpi, transcript abundance of genes related to an adaptive immune response (e.g., MHC IIB) and inflammation (Dryad Repository) was elevated. GO terms related to cellular respiration were overrepresented among down‐regulated genes (Dryad Repository). More specifically, mitochondrial cytochrome c oxidase subunits I and III (*cox1* and *cox3*) and cytochrome b (*cytb*) all had reduced transcript abundances (Dryad Repository). The full lists of overrepresented GO terms and DEG for the European eel are available from the Dryad Repository.

## 4 DISCUSSION

*Anguillicola crassus* is a parasitic swim bladder nematode native to the Japanese eel (*A. japonica*) that was first detected in the European eel (*A. anguilla*) population approximately 35 years ago (Kirk, 2003). Infection intensities measured in natural populations (Audenaert et al., 2003; Gérard et al., 2013; Heitlinger et al., 2009; Knopf, 2006; Münderle et al., 2006) and in experimental individuals several weeks after an infection was established (Knopf & Lucius, 2008; Knopf & Mahnke, 2004) indicate greater susceptibility by the novel host, the European eel. The population of the European eel has undergone catastrophic declines (Bornarel et al., 2017; Diekmann et al., 2019; ICES, 2018), and *A. crassus* infections have been implicated (Drouineau et al., 2018; Sures & Knopf, 2004). We found similar infection intensities in the two eel species 23 days after experimental infection, at which time larval parasites had finished migrating to the swim bladder, and we did not find any dead larvae. Although our sample size was small, our findings support the observations at 25 dpi by Weclawski et al. (2013) and indicate that the different abilities of the two eel species to clear an infection do not manifest early after infection, and may only become apparent after more advanced developmental stages of the parasite are present. The low number of parasites in the swim bladders we observed at 3 dpi in both eel species indicates that a considerable proportion of larvae was still migrating toward the swim bladder.

We found almost five times as many DEG in infected European eels than in infected Japanese eels. The difference in expression between species was most pronounced during the parasite migration phase (3 dpi) but remained large after parasites were established in the swim bladder wall (23 dpi). In both species, more genes were differentially expressed during the larval migration than after establishment, although this temporal difference was much more pronounced for the European eel. This could indicate that the tissue damage caused by the migrating parasites is more problematic for both eel species than the presence of larvae in the swim bladder wall. We cannot fully exclude the possibility that, although all individuals were handled identically, the infection procedure affected experimentally and sham‐infected individuals differently and that, at 3 dpi, recovery from stress of handling contributed to the overall greater number of DEG.

In the Japanese eel, immune system processes were only weakly affected, and only at 3 dpi. Those processes that were affected did not indicate that the Japanese eel attempted to clear the infection. Reduced abundance of *cd40* transcripts might indicate nonresponsiveness of the immune system during *A. crassus* migration to the swim bladder. CD40 is essential for initiating a T cell‐dependent adaptive immune response. By providing a costimulatory signal, it leads to activation, proliferation, and differentiation of lymphocytes and induces maturation of dendritic cells during interaction with T cells. The absence of costimulation induces immunological tolerance rather than immunity (Elgueta et al., 2009; Quezada, Jarvinen, Lind, & Noelle, 2004). At 23 dpi, there was no evidence for an immune response. However, only a small proportion of DEG could be annotated. While this may indicate that some species‐specific genes could be involved in the response, we do not expect that it would cause an immune response to remain undetected because the fish immune system is very similar to the well‐described mammalian immune system (Alvarez‐Pellitero, 2008; Buchmann, 2012).

Similarly to the infection intensities, we and Weclawski et al. (2013) observed, this lack of response suggests that an early and efficient immune response does not contribute to lower susceptibility observed in the Japanese eel. An important consideration is that most studies have only inferred the immune response based on infection intensities and proportions of dead *A. crassus* (Knopf & Mahnke, 2004; Weclawski et al., 2013). Other studies have assessed a response based on antibodies in the presence of adult *A. crassus*, but antibodies may not be crucial for reducing infection intensities in this system (Knopf & Lucius, 2008; Nielsen, 1999). Large numbers of dead *A. crassus* larvae in the Japanese eel were reported after adult *A. crassus* had appeared (Heitlinger et al., 2009; Weclawski et al., 2013). Taken together, there is no evidence that would suggest that the Japanese eel mounts an immune response in the early stages of first infection, and a response to any parasitic stage, including larvae that have halted development (Knopf & Mahnke, 2004), may only occur after adult stages are present in the swim bladder. However, infection with larvae that were exposed to a sublethal dose of radiation which impaired their development to adulthood led to encapsulation several weeks postinfection (Knopf & Lucius, 2008).

Infected European eels underwent gene expression changes in not only more genes, but in a wider range of processes that included immune response, renal function, and energy generation. In the Japanese eel, none of the differentially expressed genes were associated with renal function or energy budget, although erythropoiesis (production of red blood cells) may have been initiated at 3 dpi. This adds to previous findings in amphibians (Ellison et al., 2015; Eskew et al., 2018; Poorten & Rosenblum, 2016), mammals (Davy et al., 2017; Field et al., 2015), and even honeybees (Zhang et al., 2010) and indicates that a stronger response to an invasive parasite in susceptible host species than in resistant host species may be a general pattern in the response to parasites as diverse as fungi, mites, and helminths. One important consideration is that this and other studies have focused on one or few time points during infection. Resistant hosts may produce a strong response at an as yet unstudied time point during infections that lead to reduction and clearance of parasites. In contrast, susceptible hosts may respond inadequately throughout the infection.

Immune genes that were differentially expressed in the European eel indicated that eels produced an inflammatory response to *A. crassus* throughout the experiment. This did not appear to induce an adaptive immune response early during the infection because transcript abundance of genes encoding MHC IIA, TCR α, and immunoglobulin chains was reduced at 3 dpi. The abundance of *Mhc IIB* transcripts was elevated following the establishment of parasite larvae in the swim bladder wall, which may indicate that the adaptive immune response was activated at 23 dpi. The activation of immune responses was not reflected in lower infection intensities in the European eel compared with the Japanese eel, suggesting that the early response of the European eel is ineffective. Considering that the Japanese eel did not initiate an immune response this early after infection, the timing of the response by the European eel may also be inappropriate and contribute to its inefficiency. A previous study found considerably more immune genes to be differentially expressed in the swim bladder of naturally infected European eels (Schneebauer et al., 2017). There are several reasons that might account for the differences between studies. First, we experimentally infected the eels once with a moderate number of *A. crassus* larvae while Schneebauer et al. (2017) used naturally infected wild‐caught eels that contained parasites at all stages of the life cycle including adults. The European eel was previously shown to produce antibodies against adult *A. crassus* (Knopf & Lucius, 2008; Nielsen, 1999), thus the presence of adult parasites may enhance the immune response, as discussed above for the Japanese eel. Second, the response to an infection differs among organs (Bracamonte et al., 2019; Huang et al., 2016; Robledo et al., 2014). If an immune response is induced locally but not systemically, it would only be detectable in the infected organ. And third, infected and control yellow eels of Schneebauer et al. (2017) originated from different geographic locations and were likely exposed to differing parasite communities (Gérard et al., 2013; Kennedy, 2001). The immune response might therefore be influenced by the prevailing parasite community rather than the presence of a single parasite species (Huang et al., 2016; Stutz, Schmerer, Coates, & Bolnick, 2015). Our eels were kept within a recirculation system presumably free of parasites. We did not screen for additional parasites, but any other parasites present would have been homogenized throughout the system and therefore equally exposed all eels in the experiment.

Similarly to susceptible amphibians and mammals, the European eel modified the expression of genes involved in energy generation, suggesting that an infection affects maintenance of homeostasis. Transcript abundance of *cpox* which encodes an enzyme of the heme biosynthesis pathway (Layer, Reichelt, Jahn, & Heinz, 2010) was reduced at 3 dpi. This contrasts with our previous finding of increased ferrochelatase expression in the spleen of infected European eels (Bracamonte et al., 2019). Heme is an essential part of hemoproteins such as hemoglobin and cytochromes. The expression of hemoglobin, the oxygen carrier of red blood cells, is affected by *A. crassus* infections, though after the appearance of adult parasites (Fazio et al., 2009). Here, we found reduced transcript abundance of several cytochromes of the respiratory chain at 23 dpi. This may be a consequence of earlier (3 dpi) reduction of heme biosynthesis. Cell respiration provides energy and its reduction in infected European eels could lead to poor performance during energetically costly activities (Palstra et al., 2007).

Immune responses carry energetic and physiological costs, and excessive and inappropriate regulation and timing of the immune response can cause tissue damage, can interact with maintenance of homeostasis or reproduction, and can lead to fitness loss and even host death even if the infection is cleared (Graham, Allen, & Read, 2005; Lochmiller & Deerenberg, 2000; Sheldon & Verhulst, 1996). Reducing an immune response and its associated costs can therefore be beneficial for the organism. Stated another way, tolerating infections, that is, mitigating the negative effect of an infection on host health (Raberg, Graham, & Read, 2009), may be more beneficial than clearing them if the self‐inflicted damage caused by an immune response outweighs the costs of bearing an infection (Graham et al., 2005; Read, Graham, & Raberg, 2008). Considering that we infected eels once with a moderate number of *A. crassus* and we sampled before the blood‐feeding parasitic stages appeared, destroying and degrading nematode tissue might be too costly at this stage of infection. The inappropriate immune response that we observed in the European eel compared with the Japanese eel may have promoted the disruption of the nonimmune processes. This may then translate into higher health and fitness costs despite the absence of differing infection intensities at this early stage of infection. However, blood‐feeding adult *A. crassus* and continuous exposure, as seen in wild eels (Heitlinger et al., 2009), might cause more damage and trigger a noticeable protective immune response in the Japanese eel.

### 4.1 Limitations of the study

Our study provides a robust experimental comparison of both species under identical conditions, but some limitations of the study hinder a more complete understanding of the response. First, the number of DEG that were annotated was always lower than 50%, albeit similar for the European eel at both time points and for the Japanese eel at 3 dpi. The value was much lower for the Japanese eel at 23 dpi. This is a common problem in RNA‐seq studies and fundamentally hampers our understanding in nonmodel organisms (Pavey, Bernatchez, Aubin‐Horth, & Landry, 2012). Eels are fish, but evolutionarily distant from model fish species with more completely annotated genomes. Second, the possibility that the European eels were silvering, that is preparing for long‐distance oceanic migration, makes our species comparison more conservative, based on the observation that silvering leads to an overall reduction in the number of DEG compared with yellow eels (Schneebauer et al., 2017). A consequence is that our comparison may underestimate the differences between species. Although we did not measure eye diameter, we observed that European eel but not Japanese eel individuals had enlarged eyes, which is a sign of silvering (Tesch, 2003). Silvering involves morphological and physiological modifications (Durif, Dufour, & Elie, 2005; Tesch, 2003). These modifications are energetically costly and might reduce resource allocation to other functions, including the immune system. Finally, hosts and parasites undergo coevolution. Ongoing differentiation and adaptation of *A. crassus* populations (Heitlinger, Taraschewski, Weclawski, Gharbi, & Blaxter, 2014; Weclawski et al., 2013) may limit the suitability of using infections with the European parasite population as null model for the Japanese eel responses, although fitness parameters and overall gene expression of adults do not differ between Asian and European parasites (Heitlinger et al., 2014; Weclawski et al., 2013, 2014). As with silvering (above), using European parasites means that the differences between host species that we observed were conservative, and that the European response may be even more pronounced than the Japanese, if the Japanese eel in our experiment was potentially less adapted to the parasite than the European eel was.

## 5 CONCLUSION

The European eel is undergoing catastrophic population declines, and infection with *A. crassus* could be one of the contributing factors (Drouineau et al., 2018). Based on comparison of the number and diversity of differentially expressed genes with the Japanese eel, we conclude that the European eel has not adapted to *A. crassus*. However, it may be that responses to infection soon after the parasite's introduction were much stronger than what we observed here, and that some degree of adaptation has taken place in the 35 years since introduction (approx. 3–5 eel generations). The comparison between the two species further indicates that preventing disruption of metabolic and physiological processes is imperative for reducing susceptibility. In contrast, producing an immune response immediately after first contracting the parasite may not provide sufficient benefits. While the impact of parasites on host physiology or fitness may be a better estimate of the potential threat than immune response or parasite load (Viney, Riley, & Buchanan, 2005), determining the timing and associated costs of the immune response in the native host can help to clarify the optimal defence strategy and provide a baseline for identifying possible adaptation by additional hosts.

## CONFLICT OF INTEREST

The authors declare that they have no competing interests.

## AUTHOR CONTRIBUTIONS

SEB, MTM, and KK designed the study. SEB and KK performed the experiment. SEB, PRJ, and MTM analyzed the data. All authors contributed to the final manuscript.

## Data Availability

Raw RNA‐seq reads were deposited in the BioProjects PRJNA419718 (European eel 3 dpi), PRJNA546508 (European eel 23 dpi), and PRJNA546510 (Japanese eel 3 and 23 dpi). Supplementary data are available from the Dryad Repository https://doi.org/10.5061/dryad.s1rn8pk3h.
