# Corrigendum

**DOI:** 10.1002/ece3.7446

**Published:** 2021-05-10

In “Gene expression response to a nematode parasite in novel and native eel hosts,” which was published in volume 9 issue 23, December 2019, the value in Table 2 for the Down‐regulated Total DEG column for European eel was incorrect.

In the published article, the value was given as 209. However, the correct value should be 109 as seen in the corrected table below. The results and conclusions of the article are unaffected.

**TABLE 2:** Number of differentially expressed genes (DEG), DEG with annotations, and overrepresented Gene Ontology (GO) terms

Species	Dpi	Up‐regulated			Down‐regulated		
		Total DEG	Annotated	GO terms	Total DEG	Annotated	GO terms
Japanese eel	3	39	11	6	25	17	33
	23	11	2	0	12	1	6
European eel	3	233	139	134	109	49	41
	23	33	19	50	20	8	17

Abbreviation: dpi, days postinfection.
